# Insulin growth factor binding protein 2 mediates the progression of lymphangioleiomyomatosis

**DOI:** 10.18632/oncotarget.16695

**Published:** 2017-03-30

**Authors:** Xiangke Li, Xiaolei Liu, Linda Zhang, Chenggang Li, Erik Zhang, Wang Ma, Qingxia Fan, Jane J. Yu

**Affiliations:** ^1^ Department of Oncology, The First Affiliated Hospital of Zhengzhou University, Zhengzhou, Zhengzhou 450052, China; ^2^ Division of Pulmonary, Critical Care and Sleep Medicine, University of Cincinnati College of Medicine, Cincinnati, OH 45267, USA; ^3^ Program in Behavioral Neuroscience, Northeastern University, Boston, MA 02115, USA

**Keywords:** IGFBP2, estrogen, lymphangioleiomyomatosis, ERα, nuclear localization

## Abstract

Lymphangioleiomyomatosis (LAM) is a progressive pulmonary disease that almost exclusively affects women. LAM cells migrate to the lungs, where they cause cystic destruction of lung parenchyma. Mutations in *TSC1* or *TSC2* lead to the activation of the mammalian target of rapamycin complex-1, a kinase that regulates growth factor-dependent protein translation, cell growth, and metabolism. Insulin-like growth factor binding protein 2 (IGFBP2) binds insulin, IGF1 and IGF2 in circulation, thereby modulating cell survival, migration, and invasion in neoplasms. In this study, we identified that IGFBP2 primarily localized in the nucleus of TSC2-null LAM patient-derived cells *in vitro* and *in vivo*. We also showed that nuclear accumulation of IGFBP2 is closely associated with estrogen receptor alpha (ERa) expression. Furthermore, estrogen treatment induced IGFBP2 nuclear translocation in TSC2-null LAM patient-derived cells. Importantly, depletion of IGFBP2 by siRNA reduced cell proliferation, enhanced apoptosis, and decreased migration and invasion of TSC2-null LAM patient-derived cells. More interestingly, depletion of IGFBP2 markedly decreased the phosphorylation of MAPK in LAM patient-derived TSC2-null cells. Collectively, these results suggest that IGFBP2 plays an important role in promoting tumorigenesis, through estrogen and ERalpha signaling pathway. Thus, targeting IGFBP2 may serve as a potential therapeutic strategy for women with LAM and other female gender specific neoplasms.

## INTRODUCTION

Lymphangioleiomyomatosis (LAM) is a devastating pulmonary disease affecting young women. The pathogenesis of LAM is unique: histologically benign appearing smooth muscle cells arise from an unknown source and metastasize to the lungs, where they promote emphysema-like lung remodeling. About 30-40% of women with Tuberous Sclerosis Complex (TSC), a genetic disorder caused by *TSC1* and *TSC2* mutations, have radiographic evidence of LAM [1, 2]. A Mayo Clinic series of TSC patients reported that LAM was one of the leading causes of mortality in women with TSC [3]. LAM also occurs in a sporadic form (S-LAM) in women who do not have TSC, whereas somatic *TSC2* mutations were detected in abnormal cells from the lung, kidney and lymphatics, but not in adjacent normal tissues from those organs or in peripheral blood cells [4]. Numerous preclinical studies demonstrated the effect of sirolimus, a potent inhibitor of the mammalian target of rapamycin complex 1 (mTORC1), on tumor progression [5], which led to rapid clinical translation, and the result indicates that sirolimus has clinical benefit in patients with LAM [6]. In patients with end-stage LAM, lung transplantation is offered as a last resort, although recurrence of LAM has been reported in the donor lungs [7].

The reasons that LAM almost exclusively affects women remain unclear. The remarkable female predominance of LAM suggests that female hormones, including estrogen, may contribute to disease pathogenesis and progression. Both LAM cells and renal angiomyolipoma cells express estrogen receptor alpha, estrogen receptor beta, and progesterone receptor [8]. We have previously discovered that estrogen promotes the survival and lung colonization of intravenously injected *TSC2-null* rat-uterine leiomyoma-derived ELT3 cells in our preclinical mouse model of LAM [9]. Collectively, our data indicate that estrogen plays a key role in promoting the survival of disseminated TSC2-null cells during disease progression, although the precise mechanisms have remained elusive.

Insulin-like growth factor binding protein 2 (IGFBP2), an important member of the IGFBP family of proteins, binds insulin, IGF1 and IGF2 in circulation, thereby modulating cell survival, differentiation, migration, and invasion in neoplasms [10–13]. IGFBP2 also acts independently of IGF1 or IGF2 to modulate cell proliferation, invasion and survival through interaction with integrins, integrin-linked kinase and NF-κB [14]. Recently, IGFBP2 has been identified as a potential and valuable biomarker in malignancies including breast cancer [15], ovarian cancer [16], colorectal cancer [17, 18], glioblastoma [19], lung cancer [20, 21], prostate cancer [22], and gastric cancer [25]. Studies have shown that IGFBP2 plays pivotal roles in the pathogenesis of smooth muscle cell tumors [23–25]. Knockdown of IGFBP-2 blocks cell proliferation and increases the death of breast cancer MCF-7 cells [26].

Despite the role of IGFBP2 in tumorigenesis, the mechanisms underlying IGFBP2 contribution to the tumorigenic program in cancer remain unknown, especially in LAM and other hormone-dependent tumors. In this study, we tested the hypothesis that IGFBP2 is a critical mediator of estrogen-dependent survival of TSC2-null LAM patient-derived cells. We report here that estrogen increases the nuclear translocation of IGFBP2 and induces the metastatic behaviors of LAM patient-derived cells. Furthermore, depletion of IGFBP2 by siRNA decreases the growth, suppresses the migration and reduces the invasion of LAM patient-derived cells.

## RESULTS

### Nuclear accumulation of IGFBP2 is associated with ERα in LAM nodules

**Figure 1:**
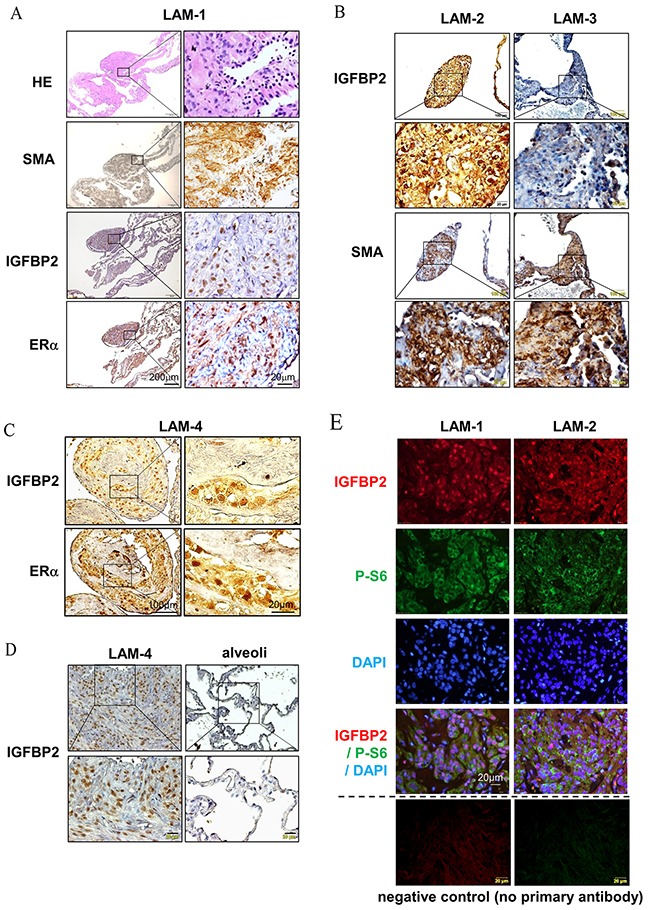
Nuclear accumulation expression of *IGFBP2* is associated with ERα in LAM nodules **(A)** Immunohistochemical staining of smooth muscle actin (SMA), IGFBP2 and ERα in pulmonary LAM nodules from one LAM subject (LAM-1). **(B)** Immunohistochemical staining of IGFBP2 and SMA in two additional LAM subjects (LAM-2 and LAM-3). **(C)** Immunohistochemical staining of IGFBP2 and ERα in additional LAM subject (LAM-4). **(D)** Immunohistochemical staining of IGFBP2 in pulmonary LAM nodules and alveolar epithelial cells in LAM-4. **(E)** Immunofluorescent staining of phospho-S6 (P-S6) (S235/236) and IGFBP2 in pulmonary LAM nodules from two LAM subjects (LAM-1 and LAM-2), and negative control with the omission of primary antibodies.

The female predominant feature of LAM suggests that E_2_ plays an important role in disease progression. Previously, we demonstrated that estradiol (E_2_) facilitates the lung metastasis of TSC2-null ELT3 cells [9]. To determine whether IGFBP2 is associated with pulmonary LAM, we assessed the expression of IGFBP2 in LAM lung nodules by immunohistochemistry. First, we identified LAM nodules from hematoxylin and eosin (HE) stained lung sections (Figure 1A). These LAM nodules cells exhibited positive smooth muscle actin (SMA)-immunoreactivity (Figure 1A, 1B). Interestingly, IGFBP2 immunoreactivity appears in the same LAM nodules whereas cells are positively stained with an ERα antibody (Figure 1C), suggesting that there is a positive correlation between high expression of ERα and nuclear accumulation of IGFBP2 in LAM lung nodules. Moreover, nuclear IGFBP2 immunoreactivity was evident in LAM cells, but not in adjacent alveolar epithelial cells (Figure 1B, 1D). Next, to examine whether nuclear accumulation of IGFBP2 is present in mTORC1-active cells, we performed double immunofluorescent staining of LAM specimens from two LAM subjects (LAM-1 and LAM-2) with IGFBP2 and phospho-S6, an indicator of mTORC1 hyperactivation. Phospho-S6-positive cells were also positively stained with IGFBP2, which primarily localized in the cell nucleus (Figure 1E, red), although phospho-S6 signal was concentrated in the cytoplasm (Figure 1E, green). Collectively, these data demonstrate that IGFBP2 accumulates in the nucleus of SMA and phospho-S6 positive LAM nodule cells in clinical specimens.

### Estrogen promotes the nuclear accumulation of IGFBP2 *in vivo*

**Figure 2:**
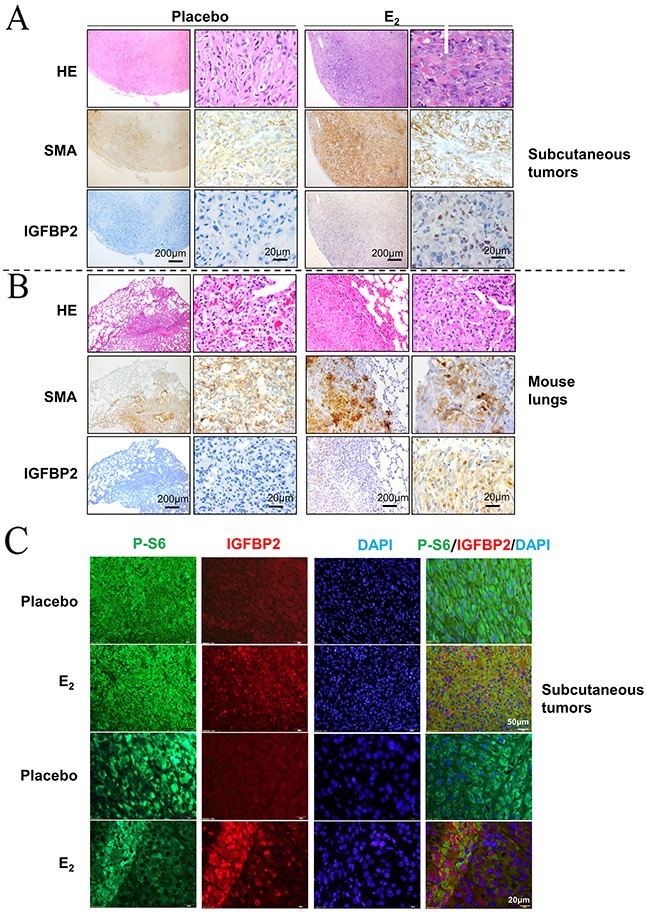
Estrogen promotes the nuclear accumulation of IGFBP2 *in vivo* **(A)** Representative immunohistochemical micrograph of IGFBP2 and SMA in xenograft tumors of ELT3 cells inoculated into female CB17 SCID mice supplemented with either E_2_ or placebo. **(B)** Representative immunohistochemical micrograph of IGFBP2 and SMA in lung metastatic lesions of ELT3 cells from mice treated with either E_2_ or placebo. **(C)** Representative immunofluorescent micrograph of IGFBP2 (red) and Phospho-S6 (green) in xenograft tumors of ELT3 cells.

To further evaluate whether nuclear-localized IGFBP2 is closely related to E_2_ stimulation *in vivo*, we performed immunohistochemical staining of IGFBP2 in xenograft tumors of TSC2-null rat-derived ELT3 cells and lung metastatic lesions of mice treated with E_2_ [9]. IGFBP2 immunoreactivity was more evident in subcutaneous tumors of Tsc2-null cells from E_2_-treated mice than placebo treatment (Figure 2A). Moreover, SMA-positive lung metastatic lesions of Tsc2-null cells from E_2_-treated mice exhibited prominent IGFBP2 immunoreactivity compared with placebo-treatment (Figure 2B). Consistent with these findings, subcutaneous tumors from E_2_-treated mice had abundant immunofluorescence of IGFBP2 (red) in phospho-S6 positive cells (green) compared with placebo treatment (Figure 2C). Collectively, our results further support that E_2_ promotes the nuclear accumulation of IGFBP2 in xenograft tumors and lung metastatic lesions of Tsc2-null rat-derived ELT3 cells *in vivo*.

### E_2_ induces nuclear translocation of IGFBP2 in LAM patient-derived cells

**Figure 3:**
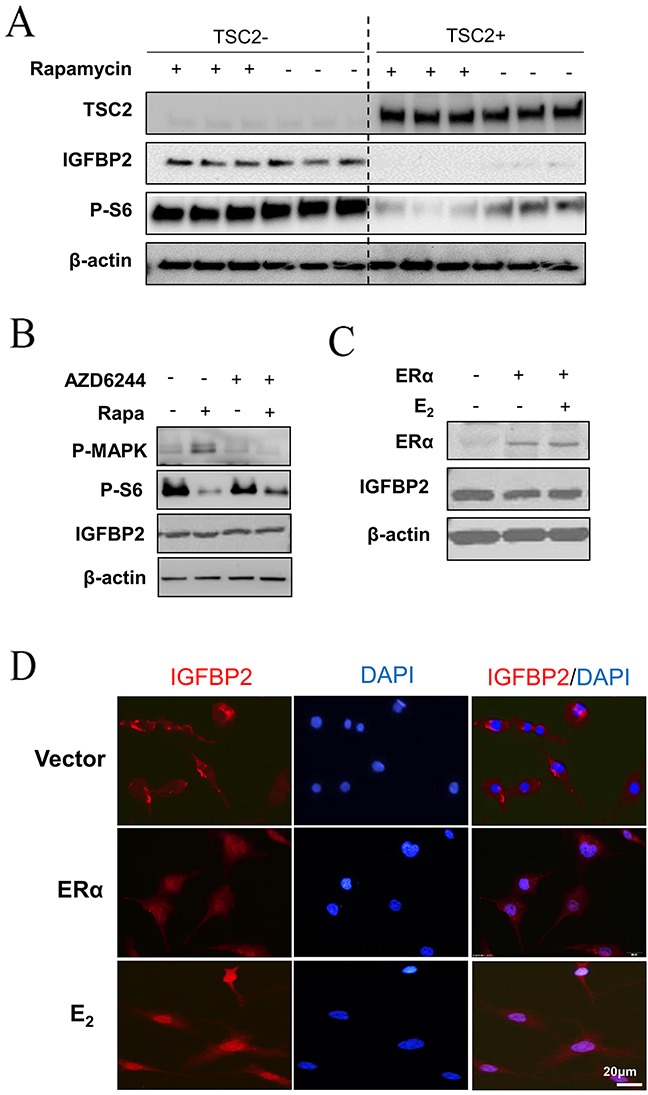
E_2_ induces nuclear translocation of IGFBP2 in LAM patient-derived cells **(A)** Immunoblot analyses of TSC2, IGFBP2, and phospho-S6 (Ser235/236) in patient-derived TSC2-null (TSC2-) or TSC2-reexpressing (TSC2+) cells treated with rapamycin (20 nM) for 24 hours. **(B)** LAM patient-derived TSC2-null cells were treated with 20 nM rapamycin, 0.5 μM AZD6244, or rapamycin plus AZD6244 for 24 hours. Immunoblot analysis of IGFBP2, phospho-S6 (P-S6) (S235/236) and phospho-p44/42 MAPK (P-Erk1/2) (Thr202/Tyr204). **(C)** ERα and IGFBP2 in LAM patient-derived TSC2-null cells transfected with ERα. β-actin represents a loading control. **(D)** Representative immunofluorescent micrograph of IGFBP2 (red). Nuclei were stained with DAPI (blue) in LAM patient-derived TSC2-null cells transfected with ERα and received E_2_ for 24 hours.

Our *in vivo* findings reveal the close relationship between the nuclear accumulation of IGFBP2 and E_2_ in TSC2-null cells. In a separate experiment, we found that TSC2-null LAM patient-derived cells expressed much higher levels of IGFBP2 compared with TSC2-reexpresing cells (Figure 3A), indicating that TSC2 negatively regulates IGFBP2 expression. Activation of the mTORC1/S6K1 signaling pathway has been documented in pulmonary LAM cells [27–29]. Thus, we examined whether mTORC1 mediates the expression of IGFBP2 in LAM patient-derived cells. Cells were treated with 20 nM rapamycin, an mTORC1 inhibitor, for 24 hours. Immunoblot analysis showed that rapamycin treatment prominently decreased phosphorylation of S6 (S235/236), but had no effect on the expression of IGFBP2 (Figure 3A), indicating that TSC2 negatively regulates IGFBP2 expression in an mTORC1-independent manner. We also found that AZD6244, an MEK1/2 inhibitor, drastically weakened the levels of phospho-p44/42 MAPK (Thr202/Tyr204), but did not affect the protein levels of IGFBP2, when employed singly and in combination with rapamycin (Figure 3B). To determine whether ERα mediates the action of E_2_ on IGFBP2 localization, we overexpressed ERα in LAM patient-derived cells. The levels of ERα protein were higher in ERα transfected cells relative to empty-vector control. However, the levels of IGFBP2 protein were not affected by ERα overexpression (Figure 3C). Using confocal microscopy, we found that 10 nM E_2_ induced nuclear accumulation of IGFBP2 in TSC2-null cells expressing ERα relative to control group (Figure 3D), indicating that E_2_ and ERα signaling axis mediates the nuclear translocation of IGFBP2.

### Depletion of IGFBP2 reduces the metastatic potentials of LAM patient-derived cells *in vitro*

**Figure 4:**
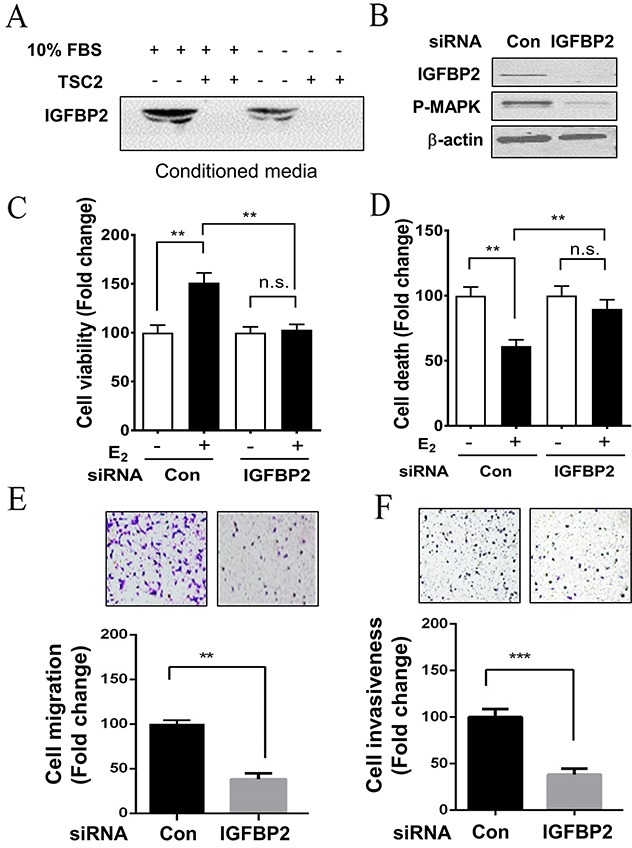
Depletion of IGFBP2 decreases the survival and metastatic potential of LAM patient-derived cell *in vitro* **(A)** Secreted levels of IGFBP2 were assessed using immunoblot analysis in concentrated conditioned media samples from TSC2-null (TSC2-) and TSC2- reexpressing (TSC2+) cells. **(B)** TSC2-null cells were transfected with IGFBP2 siRNA or control siRNA for 24 hours, and then harvested for functional assays. The protein levels of IGFBP2 and phospho-MAPK (P-MAPK) were assessed using immunoblot analysis. **(C)** Cell viability was examined using MTT assay. **(D)** Cell survival was examined using PI exclusion assay. **(E)** Cell migration was quantified using Boyden chamber migration assay. **(F)** Cell invasiveness was examined using Matrigel invasion. Mean ± SD, *p<0.05, **p<0.01, Student's *t* test.

Because IGFBPs are secretory proteins, we first examined the protein levels of IGFBP2 in conditioned media samples from LAM patient-derived cells. TSC2-null cells secreted abundant IGFBP2 protein under 10% FBS or serum-free (0% FBS) conditions compared with TSC2-expressing cells (Figure 4A). To determine if IGFBP2 has any biologic consequences in LAM patient-derived TSC2-null cells, IGFBP2 was knocked down by siRNA transfection in LAM patient-derived cells (Figure 4B). Interestingly, IGFBP2 depletion led to decreased phosphorylation of p44/42 MAPK (Figure 4B). E_2_ treatment enhanced the proliferation of LAM patient-derived TSC2-null cells by 50% compared to the control group (p<0.01). Moreover, abrogation of IGFBP2 markedly inhibited the E_2_-induced proliferation of LAM patient-derived TSC2-null cells by 33% (p<0.01), compared to the control group (Figure 4C). Furthermore, E_2_ treatment decreased the death of LAM patient-derived TSC2-null cells by 40% compared with the control group (p<0.01). Importantly, molecular depletion of IGFBP2 using IGFBP2 siRNA attenuated the effect of E_2_ on the growth of LAM patient-derived cells (Figure 4D). We next determined whether IGFBP2 is a key mediator of the migration of TSC2-null cells. LAM patient-derived TSC2-null cells transfected with IGFBP2 siRNA exhibited decreased migration compared with empty vector transfection (p<0.01) in a transwell assay *in vitro* (Figure 4E). In a Matrigel invasion assay, which detects the ability of cells invading a basement membrane matrix, LAM patient-derived TSC2-null cells in the control group passed through the transwell membrane more frequently and exhibited a greater invasive capability than LAM patient-derived TSC2-null cells transfected with IGFBP2 siRNA (p<0.001) (Figure 4F). Together, our data indicates that IGFBP2 plays an important role in promoting the growth, migration and invasion of TSC2-null cell *in vitro*.

## DISCUSSION

LAM is a devastating female predominant pulmonary disease characterized by the accumulation of abnormal smooth muscle-looking cells in the lung parenchyma and severe emphysema-like lung destruction that can lead to respiratory failure and mortality [30–33]. The high gender specificity of LAM indicates that circulating female steroid hormones including estradiol contributes to LAM pathogenesis and progression. We previously reported that estradiol enhanced the survival of tuberin-null rat uterine leiomyoma-derived ELT3 cells and promoted lung metastasis of these cells in a spontaneous metastatic mouse model [9]. In this study, we identified that IGFBP2 expression is a critical mediator of metastatic potentials of LAM patient-derived cells. Importantly, IGFBP2 accumulation is prominent in the nucleus of LAM cells *in vitro*, in xenograft tumors and lung metastatic lesions of Tsc2-null ELT3 cells in SCID mice treated with estradiol, and in clinical specimens of pulmonary LAM. Additionally, the nuclear accumulation of IGFBP2 is closely associated with ERα expression. Molecular and functional analyses of IGFBP2 of LAM patient-derived TSC2-null cells may provide insights into the biology of LAM pathogenesis. Depletion of IGFBP2 by siRNA reduced cell proliferation, enhanced apoptosis, and decreased migration and invasion of TSC2-null LAM patient-derived cells. More interestingly, depletion of IGFBP2 markedly decreased the phosphorylation of p44/42 MAPK in LAM patient-derived TSC2-null cells.

As previously reported, numerous implications suggested that there is a strong link between the ERα status and IGFBP-2. ERα is generally recognized as an important driver of hormone-responsive breast cancer. IGFBP2, having been widely accepted as one of the estrogen-responsive genes, appears to be involved in the maintenance of ERα and is regulated by E_2_ administration in ERα-positive breast carcinoma cell lines [34], rat hippocampus [35] and adult articular cartilage [36]. The transcriptional and protein synthesis levels of IGFBP2 are steroid-dependent, being regulated by steroid hormones, including estradiol and progesterone, in cultured human endometrial stromal cells [37]. Importantly, studies also demonstrated that knockdown endogenous IGFBP2 decreases the expression of ERα and, conversely, that the addition of exogenous IGFBP2 increases the protein levels of ERα. These studies suggest that there may be a positive feedback loop where ERα stimulates IGFBP2 production, and IGFBP2 influences the expression of ERα.

Additionally, LAM tissue and LAM-patient cells both exhibit ERα positive expression, nevertheless, there is no clear conclusion whether an association exists between the expression of ERα and IGFBP2, or if these molecules are involved in E_2_-facilitated lung colonization and metastasis in LAM. In previous studies, we have shown that E_2_ promotes growth and shortens the survival of immunodeficient mice inoculated with Tsc2-null ELT3 cells. Importantly, we identified E_2_-indcued nuclear accumulation of IGFBP2 in xenograft tumors and lung metastatic lesions from mice injected with ELT3 cells. We also showed that nuclear accumulation of IGFBP2 was evident in LAM lung nodules, and was likely associated with ERα levels in the same nodules.

IGFBPs are secreted proteins that bind to circulating IGF-1 [38]. It has been reported that mTORC2 regulates IGFBP3 expression [39]. Recently, Ding et al. found that IGFBP5 expression and secretion were elevated in TSC2-null cells and regulated by mTORC1, and secreted IGFBP5 acts as a potent inhibitor of IGF-1 signaling pathway [40]. Beyond its classical IGF-dependent functions, IGFBP2 exerts IGF-independent effects through interactions with the extracellular matrix, cell surface or receptors [41]. Thus, understanding the events that lead to its nuclear localization has provided insights into its role and the mechanisms behind its activation of processes involved in the promotion of tumorigenesis [10, 42]. Abrogation the expression of IGFBP-2 in experimental models of cancer types would significantly inhibit cell proliferation, migration and invasion [43]. We speculate that a novel mechanism of action for IGFBP-2 is dependent-nuclear translocation. Yet, the precise mechanism by which IGFBP2 translocate into the nucleus remains elusive in LAM.

In this study, our data confirm that IGFBP2 localizes to the cell nucleus in both LAM tissue and mouse models. E_2_ induced IGFBP2 nuclear translocation in LAM patient-derived TSC2-null cells transfected with ERα. This study demonstrates, for the first time, that nuclear localization of IGFBP2 is mediated by E_2_ and ERα signaling in pulmonary LAM. Importantly, molecular depletion of IGFBP2 expression using siRNA suppressed cell proliferation, enhanced cell death, decreased migration and invasion in LAM patient-derived TSC2-null cells. Abrogation of IGFBP2 also decreased phosphorylation of p44/42 MAPK; however, rapamycin and AZD6244 treatment did not affect the expression of IGFBP2 in TSC2-null cells. Collectively, these results indicate that IGFBP2 may exert profound effects on the progression of LAM via the phospho-p44/42 MAPK pathway. We anticipate that E_2_ and ERα play an important role in promoting tumorigenesis and disease progression via IGFBP2. Thus, targeting IGFBP2 may serve as a potential therapeutic strategy for women with LAM and other gender specific neoplasms.

## MATERIALS AND METHODS

Human samples: Lung tissues from patients with LAM were obtained from the National Disease Research Interchange (NDRI).

### Cell culture and reagents

The LAM patient-derived cells including the immortalized TSC2-null human cell line, the corresponding TSC2-addback control cell line [44, 45], and the Eker rat uterine leiomyoma-derived TSC2-null cells (ELT3) [46, 47], were cultured in IIA complete medium supplemented with 10% FBS. Before E_2_ stimulation, cells were starved overnight in serum-free and phenol red-free IIA media. If indicated, cells were also incubated with 17-β-estradiol (10 nM, Sigma-Aldrich), rapamycin (20 nM, Biomol), and/or AZD6244 (500 nM, Selleckchem).

### Preparation of conditioned media

2×10^5^ LAM patient-derived cells or TSC2-reexpressing cells were seeded in 6-well plate with 2 mL IIA complete medium supplemented with 10% FBS. When cells became 70% confluent, medium was replaced with IIA serum-free (0% FBS). 24 hr later, medium was collected and centrifuged at 2,000 rpm at 4°C for 10 min. Supernatant was transferred to Amicon Ultra-4 Centrifugal Filter Unit with Ultracel-10 membrane (10 kDa cutoff) (EMD Millipore) and concentrated.

### Immunohistochemistry and immunofluorescent staining

Paraffin-embedded 5 mm tissue sections were deparaffinized, and antigen retrieval was performed using sodium citrate retrieval solution (Sigma). Immunohistochemistry was performed using the SuperPicture™ 3rd Gen IHC Detection Kit (Life Technologies); sections were stained by incubation with primary antibodies and biotinylated secondary antibodies, counterstained with hematoxylin and mounted in Histomount. For immunofluorescent staining, sections were blocked with 10% donkey serum (Sigma), incubated with primary antibodies and respective secondary antibodies, counterstained with DAPI (Sigma), and mounted with CC/Mount (Sigma). Negative control for immunofluorescent staining was performed by omitting primary antibodies.

### Protein extraction and immunoblot analysis

Cells were rinsed once using cold 1×PBS buffer, lysed in RIPA buffer (Thermo Fisher Scientific) supplemented with protease inhibitor cocktail and phosphatase inhibitor cocktail (Sigma) and centrifuged at 14,000 rpm, 4°C for 15 minutes. Total cellular protein was analyzed by SDS-PAGE using 4–12% NuPAGE Gel (Invitrogen), transferred to nitrocellulose membrane and blocked with 5% Bovine Serum Albumin (Sigma) for 1h at room temperature. Immunoblotting was performed by using primary antibody and respective secondary antibodies, and detected using Supersignal West Pico Chemiluminescent substrate (Thermo Scientific). Antibodies were procured from: Phospho-MAPK (T202/Y204), Phospho-S6 (S235/236) (Cell Signaling Technology); IGFBP2, ERα, tuberin (Santa Cruz), and beta-actin (Sigma).

### Immunofluorescent staining

Cells were plated on glass coverslips, cultured with serum-free medium overnight, and then stimulated with control or E_2_ (10 nM) for 24 h. Cells were fixed with 4% paraformaldehyde for 15 min, blocked in 1% BSA/PBS/Tween 20 (0.05%) for 30 min, incubated with primary antibody overnight at 4°C, and then respective secondary antibodies for 1 hour. Negative control for the staining was performed with the omission of primary antibodies. Images were captured using an IX73 Olympus Inverted Microscope System (Olympus).

### Transient DNA transfection

IGFBP2-siRNA or Control-siRNA (100 nM, Santa Cruz) was transfected into cells using Trans IT-TKO reagent (Mirus, Madison, WI, USA). pcDNA-ERα was transfected into cells using Lipofectamine® 3000 (Invitrogen). Cells were harvested 24–72 h post cell transfection for biochemical assays.

### Migration and invasion assay

Cells transfected with IGFBP2-siRNA or control-siRNA for 24 h were seeded into 6.5 mm Transwell® plates (8.0 μm Pore Polycarbonate Membrane Inserts, Corning) or 24-well BioCoat™ Matrigel®Invasion upper Chamber (8.0 μm PET Membrane, BD Biosciences). The lower chamber was filled with culture media containing 10% FBS. 24 h post-seeding, non-migrating or non-invading cells from the upper surface of membrane were removed, stained with crystal violet, and quantitated using an inverted light microscope (Nikon). The lower surface of the membrane was dissolved in methanol; fluorescence was read at 540 nm using a SynergyTM HTX Multi mode reader (BioTek).

### Statistical analyses

All experiments were performed at least three times; data is presented as means ± SEM and evaluated using GraphPad Prism 6.0 (GraphPad Software). Student's *t* test was used to determine differences between two groups. P value < 0.05 was considered statistically significant.
